# Early detection of breast cancer based on gene-expression patterns in peripheral blood cells

**DOI:** 10.1186/bcr1203

**Published:** 2005-06-14

**Authors:** Praveen Sharma, Narinder S Sahni, Robert Tibshirani, Per Skaane, Petter Urdal, Hege Berghagen, Marianne Jensen, Lena Kristiansen, Cecilie Moen, Pradeep Sharma, Alia Zaka, Jarle Arnes, Torill Sauer, Lars A Akslen, Ellen Schlichting, Anne-Lise Børresen-Dale, Anders Lönneborg

**Affiliations:** 1 DiaGenic ASA, Oslo, Norway; 2 Departments of Health, Research and Policy, and Statistics, Stanford University, Stanford, CA, USA; 3 Department of Radiology, Ullevål University Hospital, Oslo, Norway; 4 Department of Clinical Chemistry, Ullevål University Hospital, Oslo, Norway; 5 Department of Pathology, The Gade Institute, Haukeland University Hospital, Norway; 6 Department of Pathology, Ullevål University Hospital, Oslo, Norway; 7 Department of Surgery, Ullevål University Hospital, Oslo, Norway; 8 Department of Genetics, The Norwegian Radium Hospital; and University of Oslo, Faculty division, The Norwegian Radium Hospital, Oslo Norway

## Abstract

**Introduction:**

Existing methods to detect breast cancer in asymptomatic patients have limitations, and there is a need to develop more accurate and convenient methods. In this study, we investigated whether early detection of breast cancer is possible by analyzing gene-expression patterns in peripheral blood cells.

**Methods:**

Using macroarrays and nearest-shrunken-centroid method, we analyzed the expression pattern of 1,368 genes in peripheral blood cells of 24 women with breast cancer and 32 women with no signs of this disease. The results were validated using a standard leave-one-out cross-validation approach.

**Results:**

We identified a set of 37 genes that correctly predicted the diagnostic class in at least 82% of the samples. The majority of these genes had a decreased expression in samples from breast cancer patients, and predominantly encoded proteins implicated in ribosome production and translation control. In contrast, the expression of some defense-related genes was increased in samples from breast cancer patients.

**Conclusion:**

The results show that a blood-based gene-expression test can be developed to detect breast cancer early in asymptomatic patients. Additional studies with a large sample size, from women both with and without the disease, are warranted to confirm or refute this finding.

## Introduction

Early detection of breast cancer can improve the chances of successful treatment and recovery. To date, mammographic screening is the most reliable method to detect breast cancer in asymptomatic patients. Although highly effective, it has significant limitations, so that the development of more accurate, convenient, and objective detection methods is needed. In the absence of microcalcification, mammography often fails to detect tumors that are less than 5 mm in size, and also mammograms of women with dense breast tissue are difficult to interpret. For example, in a study of over 11,000 women with no clinical symptoms of breast cancer, the sensitivity of mammography was only 48% for the subset of women with extremely dense breasts, compared with 78% sensitivity for the entire sample of women in the study [1]. In addition, when an abnormality has been detected, further tests involving invasive steps must complement mammography to establish whether the detected abnormality is a cancer.

A vast amount of literature is already available describing the potential use of large-scale gene expression analysis in disease diagnosis, including breast cancer [2-8]. However, most published work with implications in cancer diagnosis has involved clinical samples comprising either diseased tissues or cells. Obtaining such samples for clinical purposes requires a prior knowledge of both their presence and their location in the body. A gene-expression-based test to detect cancers that does not rely upon the availability of tissues or cells from the diseased area has not yet been described.

It has recently been suggested that circulating leukocytes can be viewed as scouts, continuously maintaining a vigilant and comprehensive surveillance of the body for signs of infection or other threats, including cancer [9]. In line with this view, we show that peripheral blood can be used to develop a gene-expression-based test for early detection of breast cancer. The rationale for using blood cells as monitors for a malignant disease elsewhere in the body is based on the hypothesis that a malignant growth will cause characteristic changes in the biochemical environment of blood. These changes will affect the expression pattern of certain genes in blood cells.

In this pilot study, we have analyzed gene-expression patterns in peripheral blood cells of women diagnosed with breast cancer and women with no signs of this disease. We have identified a panel of genes with distinct expression patterns in cancer versus noncancer samples. The results indicate that breast cancer causes characteristic changes in the biochemical environment of blood already during early stages of disease development. Blood cells sense and respond to the change by decreasing the expression of genes involved in protein synthesis and increasing the expression of defense-related genes. We show that the expression pattern of the identified genes can be used to discriminate and predict the class of breast cancer and non-breast-cancer samples with high accuracy. Our findings should pave way for the development of a blood-based gene-expression test for early detection of breast cancer.

## Materials and methods

### Blood samples

Blood samples were collected from donors with their informed consent under an approval from Regional Ethical Committee of Norway (331-99-99138). All donors were treated anonymously during analysis. Blood was drawn from women with a suspect initial mammogram, prior to any knowledge of whether the abnormality observed during first screening was benign or malignant. In all cases, the blood samples were drawn between 8 a.m. and 4 p.m. From each woman, 10 ml blood was drawn by skilled personnel either in vacutainer tubes containing ethylenediaminetetraacetic acid (EDTA) as anticoagulant (Becton Dickinson, Baltimore, MD, USA) or directly in PAXgene™ tubes (PreAnalytiX, Hombrechtikon, Switzerland). Blood collected in EDTA-containing tubes was immediately stored at -80°C, while PAX tubes were left overnight at room temperature and then stored at -80°C until use.

### Preparation of cDNA arrays

One thousand four hundred thirty-five cDNA clones were randomly picked from a plasmid library constructed from whole blood of 550 healthy individuals (Clontech, Palo Alto, CA, USA). Based on the sequence analysis of more than 500 cDNAs, redundancy among the randomly picked clones was estimated to be about 20%. For amplification of inserts, bacterial clones were grown in microtiter plates containing 150 μl Luria Broth media with 50 μg/ml carbenicillin, and incubated overnight with agitation at 37°C. To lyse the cells, 5 μl of each culture was diluted with 50 μl dH_2_O and incubated for 12 min at 95°C. Of this mixture, 2 μl were subjected to a PCR reaction using 40 μmol of 5' – and 3' – sequencing primers in the presence of 1.5 mM MgCl_2_. PCR reactions were performed with the following cycling protocol: 4 min at 95°C, followed by 25 cycles of 1 min at 94°C, 1 min at 60°C, and 3 min at 72°C either in a RoboCycler Temperature Cycler (Stratagene, La Jolla, CA, USA) or DNA Engine Dyad Peltier Thermal Cycler (MJ Research Inc, Waltham, MA, USA). The amplified products were denatured with NaOH (0.2 M, final concentration) for 30 min and spotted onto Hybond-N^+ ^membranes (Amersham Pharmacia Biotech, Little Chalfont, UK), using a MicroGrid II workstation in accordance with the manufacturer's instructions (BioRobotics Ltd, Cambridge, UK). The immobilized cDNAs were fixed using a UV cross-linker (Hoefer Scientific Instruments, San Francisco, CA, USA).

The printed arrays also contained controls for assessing background level, consistency, and sensitivity of the assay. These were spotted at multiple positions in addition to the 1,435 cDNAs, and included controls such as PCR mix (without any insert); controls of the SpotReport™ 10-array validation system (Stratagene), and cDNAs corresponding to constitutively expressed genes such as β-actin, γ-actin, glyceraldehyde-3-phosphate dehydrogenase, human ornithine decarboxylase and cyclophilin.

### RNA extraction, probe synthesis, and hybridization

Blood collected in EDTA tubes was thawed at 37°C and transferred to PAX tubes, and total RNA was purified in accordance with the supplier's instructions (PreAnalytiX). From blood collected directly in PAX tubes, total RNA was extracted in the tubes as above without any transfer to new tubes. Contaminating DNA was removed from the isolated RNA by DNAase I treatment using a DNA-free kit (Ambion Inc, Austin, TX, USA). RNA quality was determined visually by inspecting the integrity of 28S and 18S ribosomal bands after agarose-gel electrophoresis. Only samples from which good-quality RNA was extracted were used in this study. In our experience, blood collected in EDTA tubes often resulted in poor-quality RNA, whereas blood collected in PAX tubes almost always yielded good-quality RNA. The concentration and purity of extracted RNA were determined by measuring the absorbance at 260 nm and 280 nm. From the total RNA, mRNA was isolated using Dynabeads in accordance with the supplier's instructions (Dynal AS, Oslo, Norway).

Labeling and hybridization experiments were performed in 16 batches. The number of samples assayed in each batch varied from six to nine. To minimize the noise due to batch-to-batch variation in printing, only the arrays manufactured during the same print run were used in each batch. When samples were assayed more than once (replicates), aliquots from the same mRNA pool were used for probe synthesis. For probe synthesis, aliquots of mRNA corresponding to 4 to 5 μg of total RNA were mixed together with oligodT_25NV _(0.5 μg/μl) and mRNA spikes of the SpotReport™ 10-array validation system (10 pg; Spike 2, 1 pg), heated to 70°C, and then chilled on ice. The probes were synthesized by reverse transcription in 35 μl reaction mix in the presence of 50 μCi [α^33^P]dATP, 3.5 μM dATP, 0.6 mM each of dCTP, dTTP, dGTP, 200 units of SuperScript II reverse transcriptase (Invitrogen, Life Technologies, Carlsbad, CA, USA), and 0.1 M DTT labeling for 1.5 hours at 42°C. After synthesis, the enzyme was deactivated for 10 min at 70°C and mRNA removed by incubating the reaction mix for 20 min at 37°C in 4 units of Ribo H (Promega, Madison, WI, USA). Unincorporated nucleotides were removed using ProbeQuant G 50 columns (Amersham Biosciences, Piscataway, NJ, USA).

The membranes were equilibrated in 4 × standard saline citrate (SSC) (1 × SSC, 0.15 M NaCl, 0.015 M sodium citrate, pH 7.0) for 2 hours at 30°C and prehybridized overnight at 65°C in 10 ml prehybridization solution (4 × SSC, 0.1 M NaH_2_PO_4, _1 mM EDTA, 8% dextran sulfate, 10 × Denhardt's solution, 1% SDS). Freshly prepared probes were added to 5 ml of the same prehybridization solution, and hybridization continued overnight at 65°C. The membranes were washed at 65°C with increasing stringency (2 × 30 min each in 2 × SSC, 0.1% SDS; 1 × SSC, 0.1% SDS; 0.1 × SSC, 0.1% SDS).

### Quantification of hybridization signals

The hybridized membranes were exposed to Phosphoscreens (super resolution) and an image file generated using PhosphoImager (Cyclone, Packard, Meriden, CT, USA). The identification and quantification of the hybridization signals, as well as subtraction of local background values, were performed using Phoretix™ software (Nonlinear Dynamics, Newcastle upon Tyne, UK). For background subtraction, the median of the line of pixels around each spot outline was subtracted from the intensity of the signals assessed in each spot.

### Data analysis

From the background-subtracted data for 1,435 genes, 1.25% of the lowest and 1.25% of the highest signals were trimmed from each membrane. Since the cDNAs with signals falling within this range varied between membranes, values of 67 cDNAs in total were removed from all membranes, and the expression data for only 1,368 genes were further analyzed. The data were normalized by dividing the value of each spot by the mean of signals in each array followed by a cube-root transformation. Supplementary Fig. 1 (left panel) (Additional file 1) shows a clear batch effect in the cube-root-normalized data (similar effects were also visible in the raw data). A simple one-way analysis of variance (ANOVA) was performed to adjust for the batch effects. Supplementary Fig. 1 (right panel) (Additional file 1) shows that the systematic batch effects were removed by the ANOVA adjustment. The batch-adjusted data were then analyzed using the nearest-shrunken-centroid method [10].

In this method, standard 'external' cross-validation is used to determine the optimal shrinkage threshold. This optimal threshold is then used with the full training set to construct the centroid. As a result, for each value of the threshold, the estimate of cross-validation error obtained is approximately unbiased for the true test-error rate.

The leave-one-out cross-validation approach was used in this work. The data were divided into *M *nonoverlapping subsets (*M *= number of unique blood samples present). The model was then trained *M*-1 times on these subsets combined, each time leaving out one of the subsets (unique blood sample) from the training data, but using only the omitted subset to compute the prediction error. The errors obtained on all parts were added together and used to compute the overall misclassification error. It is well known that leave-one-out cross-validation provides an approximately unbiased and reliable estimate of the misclassification rate that would be obtained from an independent sample of patients [11,12]. In the terminology of Ambroise and McLachlan [12], we used external cross-validation (as they recommend).

The raw and the batch-adjusted data for 1,368 genes in an Excel file is provided in Supplementary Table 1 (Additional file 2) and Supplementary Table 2 (Additional file 3).

## Results

We analyzed gene-expression patterns in 60 blood samples obtained from 56 different women (Table 1). The experiments were performed in 16 batches. To investigate the reproducibility of results, 13 samples from women with breast cancer and 23 samples from women with no breast cancer were analyzed in different batches using aliquots from the same mRNA pool, giving a total of 102 experimental samples.

**Figure 1:**
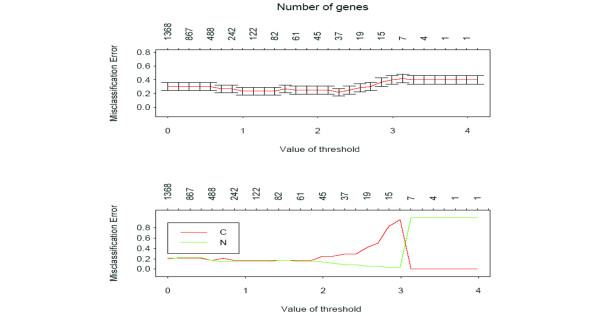
Misclassification rate as a function of threshold value and the number of genes involved. The error was calculated using the majority rule. A nondecision was counted as an error. The upper graph shows that the minimum overall misclassification error was observed at a threshold value of 2.28. The lower graph shows the profile for misclassification error for breast-cancer (C) and non-breast-cancer (N) samples as a function of threshold value and the number of genes involved.

The generated expression data was preprocessed and then analyzed by the nearest-shrunken-centroid method [10]. A standard leave-one-out cross-validation approach was used to determine the optimal amount of shrinkage threshold. Since we had 60 unique blood samples and for some of them experiments were replicated more than once, for cross-validation the data were divided into 60 nonoverlapping subsets, where each subset represented a unique blood sample and included all the replicates present in the data set. A sample was judged as correctly classified only when a majority of members in the corresponding cross-validation segment were correctly classified. The minimum overall misclassification error was observed at a threshold value of 2.28, yielding a subset of 37 genes (Fig. 1). At this threshold, 10 of the 57 samples were misclassified and 3 samples were judged nondecisions, because there was no majority for either the breast-cancer or non-breast-cancer class (Table 2). A detailed prediction result is presented in Table 1[Table T1].

The prediction was highly accurate for samples from women with early stages of breast cancer, stage 0 and stage I. Among the 14 samples representing early stages, there was one nondecision and 11 of 13 samples were correctly predicted. Five of seven stage II and one of two stage III samples were correctly predicted.

Most of the cancer samples (22 of 24) analyzed in this study were obtained from women who had cancer of ductal origin. One woman, the origin of whose cancer was not known, had a previous history of breast cancer and at the time of blood collection the cancer had spread to supraclavicular and infraclavicular nodes. Another sample that did not belong to the ductal group was obtained from a woman who had invasive lobular carcinoma in one breast and a tubular adenocarcinoma in the other. Unlike ductal carcinoma, which originates from cells lining ducts, lobular carcinoma originates from cells lining lobules. Both samples were incorrectly predicted. It is possible that cancer of other than ductal origin affects the expression pattern of the selected 37 genes in blood cells differently than ductal carcinomas.

Seventeen of 19 samples obtained from women with a suspect first mammogram were correctly predicted (Table 1, subgroup A2), indicating the expression profile of the selected 37 genes to be highly efficient in discriminating between cancerous and noncancerous breast abnormalities. In two samples, we were not able to make any diagnostic decision.

Among the 17 samples from women with no reported breast abnormality, 13 were correctly predicted (Table 1, subgroup A3). These included samples from breast-feeding women as well as those drawn at different times in the menstrual cycle from one woman. However, the three samples from pregnant women and a sample from a woman with acute bacterial infection at the time of blood collection were all incorrectly predicted. The woman with acute bacterial infection was, in addition, chronically infected with Epstein–Barr virus. It is known that both pregnancy and chronic infection may elicit responses that can mimic breast cancer. During late pregnancy, similar to breast cancer, cells of mammary epithelial buds divide to form ducts infiltrating breast stroma and build a local blood supply. Also, both breast cancer and chronic infections are known to induce inflammatory responses in the body.

We also calculated the misclassification error, taking an average of the class probability for each sample in all 60 cross-validation segments as compared with our previous approach in which a sample was judged as correctly classified only when a majority of members in the corresponding cross-validation segment were correctly classified. Thus, each segment represented an average class probability for each sample, and we predicted each sample to the class with the highest average probability. The main purpose of adopting this approach was to be able to make a unanimous decision with respect to class membership. The minimum error rate using the average-class approach was obtained at a threshold value of 2.42 and involved a subset of only 25 genes, giving a further reduction of 12 genes (Supplementary Fig. 2) (Additional file 4). Also, 10 (7 breast cancer and 3 non-breast-cancer samples) of the 60 samples were misclassified, which is a slightly better result than that obtained with 37 genes, where there were 3 nondecisions (Supplementary Fig. 3) (Additional file 5).

**Figure 2:**
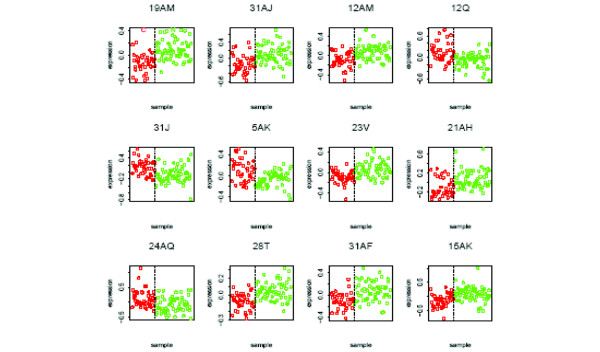
Relative expression of 13 predictive genes with the highest scores in breast-cancer and non-breast-cancer samples. Red circles represent samples from women with breast cancer and green circles represent samples from women with no signs of breast cancer. The number on the upper axis represents the position ID of predictive genes in the array (Table 3).

Table 3 shows the shrunken *t*-statistic scores of the selected 37 predictive genes for comparing breast-cancer class to non-breast-cancer class, the genes in the public databases to which they show sequence similarity, and their putative biological function. The relative expression of 12 predictive genes with highest scores is presented in Fig. 2. The majority of the predictive genes (29 of 37) had a decreased expression (positive score) in the samples from breast cancer patients. The identity of predictive genes was determined by partially sequencing the corresponding spotted cDNA clones and searching for gene similarities in public databases.

Sequence analysis revealed that 8 of 35 predictive genes contained redundant information. Since the arrayed cDNAs were derived from randomly picked clones from a library constructed from whole blood from 550 healthy individuals, we had expected a redundancy of about 20% among the selected genes. Of the 35 genes, 18 (51%) encoded ribosomal proteins. In comparison, the frequency of cDNAs representing ribosomal proteins was estimated to be only about 8% among the arrayed cDNAs. All genes encoding ribosomal proteins had reduced expression in samples from breast cancer patients, indicating a decrease in ribosome production in the blood cells of these patients. Also, genes encoding a translation elongation factor, eEF1 and RACK1 (receptor for activated C kinase), were expressed at a lower level in samples from cancer patients, indicating reduced protein translation activity in these samples. RACK1 plays a key role in the joining of 60S and 40S subunits into a functionally active 80S ribosome complex [13].

Among the eight predictive genes with increased expression in samples from breast cancer patients, two encoded histone replacement protein H3.3, which is thought to be involved in chromatin remodelling [14], and six encoded proteins that may play a role in defense-related functions. Four genes with increased expression encoded ferritin and calgranulin B. Ferritin is involved in intracellular storage and sequestration of iron. Increased expression of ferritin has been shown to reduce the accumulation of reactive oxygen species in response to oxidant challenge in HeLa cells [15]. Calgranulin B is expressed by blood cells both during infection and during inflammation and may play a role in host defense [16]. Interferon-induced transmembrane protein 2 has been implicated in the immune response, while human granule proteoglycan peptide core is assumed to form stable complexes with proteases and other granule-localized proteins to prevent their intragranular autolysis and facilitate their concerted action extracellularly [17]. Interestingly, most predictive genes identified in this study belonged to the family of genes that exhibited altered expression in neutrophils after stimulation by nonvirulent and virulent bacterial stimuli [18,19].

## Discussion

This is a first report demonstrating that breast cancer affects gene-expression patterns in peripheral blood cells during early stages of disease development. The results presented represent an initial phase in the development of a blood-based gene-expression test for breast cancer detection. A larger number of samples, from both women with and women without the disease, should be further analyzed before the clinical efficacy of our finding can be evaluated. However, the results clearly show that by analyzing the expression pattern of selected genes in blood cells, a diagnostic test for breast cancer detection can be efficiently developed.

In the present study, we examined gene-expression patterns in peripheral blood cells as a whole, rather than specific cellular subsets. It has recently been shown that individual variations in gene-expression pattern in peripheral blood could be traced to altered relative proportions of the specific blood cell subsets [9]. If there were systematic differences in the relative proportions of peripheral blood cell types in women with breast cancer and those without this disease, such differences might explain the observed gene-expression patterns. Interestingly, Whitney and colleagues [9] found that transcripts involved in protein synthesis were over-represented in lymphocytes and monocytes as compared with granulocytes. The reduced expression of transcripts involved in protein synthesis and the increased expression of transcripts involved in defense responses in breast cancer patients may reflect a systematic shift in favor of granulocytes as compared with lymphoid cells in the peripheral blood of breast cancer patients. However, to our knowledge, no such systematic shift during breast cancer development has been reported, and the subject requires further investigation. Alternatively, changes in the expression pattern of genes involved in protein synthesis, chromatin remodelling, and defense-related genes in the blood samples of breast cancer patients may indicate systematic activation of certain blood cell subsets such as neutrophils in these patients.

Our ability to correctly assign the class of samples from women with Crohn's disease, rheumatic disease, or diabetes as non-breast-cancer suggests that breast cancer affects the expression pattern of identified predictive genes differently from some of the diseases associated with anemia and chronic inflammation. The correct prediction of two samples from a woman with ductal carcinoma *in situ *further suggested that malignant lesions, though confined within the breast duct, may induce similar changes in the expression pattern of these genes to the changes seen during the more advanced stages of breast cancer (stages I to III). However, incorrect prediction of a sample obtained from a woman with invasive lobular carcinoma and tubular adenocarcinoma and from a woman where the cancer had spread to supraclavicular and infraclavicular nodes indicates that malignancy in itself is not a prerequisite condition for the observed changes in the expression pattern of the identified predictive genes.

The efficient prediction of samples derived from patients whose cancer had not yet spread to lymph nodes shows that a blood-based gene-expression test can be developed for breast cancer detection in asymptomatic patients. As compared with existing methods, an accurate method for breast cancer detection based on peripheral blood as a clinical sample will be highly desirable because of the easy accessibility and the less invasive procedure for obtaining samples. The test could be integrated as an adjunct to already established methods and be used to improve their efficacy. For example, a blood-based gene-expression test could assist mammography in discriminating between benign and malignant breast abnormalities. It could become a part of routine screening programs, especially when the patient has an increased risk for breast cancer.

It is important that any test intended for use in breast cancer diagnosis has a low rate of both false positives and false negatives. Based on the expression pattern of identified 37 genes, the prediction achieved corresponded to a false positive rate of 0.12 and false negative rate of 0.26. Since, the main goal of this work was to see whether the information about breast cancer is present in peripheral blood samples in the form of changed gene-expression patterns, we analyzed only a limited number of gene candidates in this study. The genes analyzed corresponded to clones that were randomly picked from a plasmid library constructed from whole blood of 550 individuals. The motivation for this approach for selecting gene candidates was based on the assumption that if the expression pattern of certain genes in blood cells is affected during early stages of breast cancer, the genes affected would most likely include ones involved in cell maintenance and general metabolism. Since such genes are expressed at high level in a cell, they would be frequently represented in a cDNA library and selected preferentially when randomly picked. It is our view that expression techniques such as microarrays, where the expression of thousands of genes can be monitored simultaneously, can further be used to screen for better predictive genes and develop more accurate diagnostic models.

We envisage blood-based gene-expression tests to have the potential of becoming a versatile and powerful tool for detection of disease, including other forms of cancers. As with breast cancer, other diseases may also cause characteristic changes in the biochemical environment of blood and affect the gene-expression patterns in blood cells. Specific gene-expression-based models can then be developed and used for diagnostic purposes.

## Conclusion

The results presented show that breast cancer even during early stages of disease development affects the expression pattern of certain genes in peripheral blood cells. By identifying these genes and analyzing their expression pattern, it is possible to develop a blood-based gene-expression test for early detection of breast cancer. Additional studies with a large sample size, both from women with and without the disease, are warranted to confirm or refute this finding.

## Abbreviations

ANOVA = analysis of variance; EDTA = ethylenediaminetetraacetic acid; eEF = eukaryotic elongation factor; RACK1 = receptor for activated C kinase 1; SSC = standard saline citrate (1 × SSC, 0.15 M NaCl, 0.015 M sodium citrate, pH 7.0).

## Competing interests

PvS, NSS, HB, MJ, LK, CM, PdS, AZ, and AL are employees of DiaGenic. None of the other authors have any competing interests.

## Authors' contributions

PvS and AL conceived the experiments. PvS, AL, and NSS designed the experiments. HB, MJ, CM, AZ, LK, PdS, PvS, and AL performed the experiments. PSk, PU, ES, TS, JA, and LAA provided the samples and their clinical details. RT and NSS performed the statistical analysis. PvS wrote the paper. RT, ALBD, AL, NSS, and PSk provided helpful comments during preparation of the manuscript. All authors read and approved the final manuscript.

## Supplementary Material

Additional File 1 Supplementary Figure 1, a pdf showing batch adjustment. (Left) Normalized data before batch adjustment; (right) normalized data after batch adjustment by ANOVA. Click here for file

Additional File 2 Supplementary Table 1, an Excel file showing the raw data for 1,368 genes. C, breast-cancer class; N, non-breast-cancer class. Click here for file

Additional File 3 Supplementary Table 2, an Excel file showing the batch-corrected data for 1,368 genes. C, breast-cancer class; N, non-breast-cancer class. Click here for file

Additional File 4 Supplementary Figure 2, pdf showing misclassification rate as a function of threshold value and the number of genes involved when the error is calculated by taking an average of the class probability for each sample in all 60 cross-validation segments. The upper graph shows that the minimum overall misclassification error is observed at a threshold value of 2.42. The lower graph shows the profile for the misclassification error for breast-cancer (C) and non-breast-cancer (N) samples as a function of threshold value and the number of genes involved. Click here for file

Additional File 5 Supplementary Figure 3, a pdf showing estimated cross-validated probabilities of 60 different blood samples. Red circles represent breast-cancer class (C) and green circles represent non-breast-cancer class (N). Each sample has two probabilities, one for the breast-cancer class and the other for the non-breast-cancer class. The sample is classified in the class whose probability is >0.5. Click here for file

## Figures and Tables

**Table 1 T1:** Gene-expression patterns in 60 blood samples obtained from 56 different women

Subgroup A1: Women with breast cancer									
Sample ID	Age (y)	Stage	Histology	Grade	Size (mm)	Nodes	Comments/ other disease if present	Times assayed	Prediction (37 genes)
3	54	I	IDC	1	11	0	#	2	+
5	67	0	DCIS	2	20	0	#	3	+
7	51	II	IDC	3	20	1/7	#	2	+
8	84	II	IDC	1	22	2/2	#	2	+
15	66	I	IDC	2	15	0	Rheumatic disease	3	+
16	68	I	IDC	1	7	0	#	1	+
17	66	II	IDC	1	26	0	Epilepsy	1	-
27	48	I	IDC	2	4	0	#	2	ND
31	47	I	IDC	2	15	0	#	2	-
35	44	II	IDC	2	25	0	#	1	+
36	50	I	Multifocal IDC	1	5 × 14	0	#	1	-
38	n.a.	0	DCIS	2	9	0	#	1	+
39	65	I	IDC	1	15	0	#	1	+
40	n.a.	I	IDC	2	14	0	Psoriasis	1	+
42	71	I	IDC	1	8	0	#	1	+
44	55	III	IDC	1	35	0	#	1	+
45	63	II	IDC	3	23	0	#	1	-
48	65	IV	-	-	-	Metastases in supra- and infra- clavicular nodes	Breast cancer, 1982	1	-
49	65	I	IDC	1	11	0	Type 2 diabetes	3	+
50	69	III	ILC	2	50	2/19	#	2	-
51	50	II	IDC	2	24	0	#	2	+
53	60	II	IDC	2	23	0	#	2	+
59	63	I	IDC	1	10	0	#	2	+
60	52	I	IDC	1	3	0	#	2	+
Subgroup A2: Women with abnormal first mammography									
Sample ID	Age (y)	Breast abnormality					Comments / other disease if present	Times assayed	Prediction (37 genes)
1	44	Benign density					#	2	+
2	53	Benign microcalcifications					Encapsulated cyst in left knee	2	+
4	45	Benign density					#	2	+
11	46	Benign density					Ulcerative colitis since 1983	2	+
12	44	Benign density					#	2	+
13	50	Benign density					Type 1 diabetes	2	+
14	47	Benign microcalcifications					#	2	+
19	46	Benign density, cyst					Crohn's disease	2	+
20	n.a.	Benign density					Rheumatic disease	1	+
28	44	Benign microcalcifications					#	2	+
29	63	Benign density, cyst					Fibromyalgia	2	ND
30	46	Benign density					#	2	+
32	59	Benign tumor, fibroadenoma					#	2	+
34	45	Benign density					Type 2 diabetes	2	+
41	50	Fibrosis, benign					Size histology 60 mm	1	+
43	51	Radial scar					Size histology 10 mm	1	+
52	47	Benign density					#	2	ND
54	52	Benign microcalcifications					Cancer, large intestine, 1992	1	+
58	46	Benign density					#	2	+
Subgroup A3: Women with no reported breast abnormality									
Sample ID	Age (y)						Comments	Times assayed	Prediction (37 genes)
6	42						#	3	+
9	30						Breast feeding	2	+
10	34						Breast feeding	3	+
21	26						#	1	+
22	-						#	1	+
18*	18						Week 1	2	+
23*							Week 2	1	+
24*							Week 3	1	+
26*							Week 4	2	+
25*							Week 5	1	+
33	34						Pregnant, 8 months	3	-
37	51						Acute bacterial infection in addition to chronic Epstein–Barr virus infection	1	-
46	27						Pregnant, 6 months	1	-
47	29						Pregnant, 9 months	1	-
55	43						#	1	+
56	43						#	2	+
57	22						#	2	+

Sample detail. Stage 0, *in situ *carcinoma; Stage I, invasive carcinoma with tumor size <20 mm; Stage II, invasive carcinoma with tumor size >20-50 mm; Stage III, invasive carcinoma with tumor size >50 mm. Stage IV, cancer spread to distant parts. *, Blood samples taken on five consecutive weeks from the same woman; -, incorrectly predicted; #, no relevant information available; +, correctly predicted; DCIS, ductal carcinoma *in situ*; IDC, invasive ductal carcinoma; ILC, invasive lobular carcinoma; n.a., not available; ND, nondecision.

**Table 2:** Confusion matrix of prediction results using 37 genes^a^

True/Predicted	C	N	Error rate^b^
C	17	6	0.26
N	4	30	0.12

^a^When there was no majority for either the breast-cancer or non-breast-cancer class, the prediction was regarded as a nondecision. ^b^Total error rate = 0.18; 3 nondecisions. C, breast-cancer samples; N, non-breast-cancer samples.

**Table 3:** Details of the identified 37 predictive genes

Accession no.	Gene similarity	Putative cellular function	Position ID	Score^a^
BC000514	Ribosomal protein L13a	Ribosome production	19AM	0.8377
BC007512	Ribosomal protein L18a	Ribosome production	31AJ	0.7321
BC019093	Guanine nucleotide binding protein, beta polypeptide 2-like; RACKs (receptors for activated C kinase)	Protein translation	12AM	0.6972
BC009696	Interferon induced transmembrane protein 2	Cell – environment interaction, Immune response	12Q	-0.6962
BC047681	S100 calcium binding protein A9 (Calgranulin B)	Defence; inhibition of casein kinase II	31J	-0.6444
BC066901	H3 histone, family 3B (H3.3B)	Chromatin remodelling	5AK	-0.6394
BC034149	Ribosomal protein S3	Ribosome production	23V	0.639
AK026634	Highly similar to HUMTI227HC, mRNA for TI-227H	-	21AH	0.627
BC047681	S100 calcium binding protein A9 (Calgranulin B)	Defence; inhibition of casein kinase II	24AQ	-0.627
BC001126	Ribosomal protein S14	Ribosome production	28T	0.6231
NM_000980	Ribosomal protein L18a	Ribosome production	31AF	0.6215
AY495316	Cytochrome *c *oxidase subunit, COX 1	Mitochondrial electron transport chain	15AK	0.6112
NM_001016	Ribosomal protein S12	Ribosome production	22S	0.6102
-	-	-	20AG	0.5839
BC016378	Ribosomal protein S11	Ribosome production	8S	0.5827
AY495316	Cytochrome *c *oxidase subunit, COX 1	Mitochondrial electron transport chain	27AG	0.5729
AF077043	Ribosomal protein L36	Ribosome production	3AR	0.5699
AF346981	Mitochondrial 16S rRNA	Ribosome production	25P	0.5507
BC013857	H3 histone, family 3A	Chromatin remodelling	3T	-0.5496
M22146	Ribosomal protein S4	Ribosome production	31U	0.5176
BC016857	Ferritin, heavy polypeptide 1	Iron storage; defence against ROS	6N	-0.5134
BC053370	Ribosomal protein SA	Ribosome production	2G	0.5113
BC010165	Ribosomal protein S2	Ribosome production	2V	0.5071
BC009689	Cyclin D-type binding protein	E2F-mediated transcription	21O	0.4978
BC018641	Eukaryotic translation elongation factor 1α (eEF1A)	Protein translation	4AA	0.4974
D87735	Ribosomal protein L14	Ribosome production	19H	0.486
-	-	-	6AQ	0.4837
BC016857	Ferritin, heavy polypeptide 1	Iron storage; defence against ROS	3AB	-0.481
BC012146	Ribosomal protein L3	Ribosome production	32AM	0.4776
BC001126	Ribosomal protein S14	Ribosome production	25R	0.4759
BC006784	Ribosomal protein S14	Ribosome production	24AJ	0.4695
J03223	Human secretory granule proteoglycan peptide core	Defence (may neutralize hydrolytic enzymes)	11H	-0.4681
AY147037	Myeloid/lymphoid or mixed-lineage leukemia 5 cDNA	Chromatin remodeling and cellular growth suppression	30AP	0.4669
CD246392, EST	Agencourt_14095501 NIH_MGC_172 cDNA	-	8AK	0.4666
AY339570	Cytochrome *c *oxidase subunit, COX 1	Mitochondrial electron transport chain	2E	0.4662
U43701	Human ribosomal protein L23a	Ribosome production	8G	0.4629
AY495252	Mitochondrial 16S rRNA	Ribosome production	8AF	0.4625

The position of genes in the array is shown as well as their scores, the accession number of sequences in public databases that match them, and their known or putative cellular function. ^a^The score is a shrunken *t*-statistic for comparing breast-cancer class to non-breast-cancer class. A positive score means that expression was greater in the noncancer sample than the cancer sample; a negative score means that expression was greater in the cancer sample than the noncancer sample. -, no information available; ROS, reactive oxygen species.

## References

[B1] Kolb TM, Lichy J, Newhouse JH (2002). Comparison of the performance of screening mammography, physical examination, and breast US and evaluation of factors that influence them: an analysis of 27,825 patient evaluations. Radiology.

[B2] Bertucci F, Nasser V, Granjeaud S, Eisinger F, Adelaide J, Tagett R, Loriod B, Giaconia A, Benziane A, Devilard E (2002). Gene expression profiles of poor-prognosis primary breast cancer correlate with survival. Hum Mol Genet.

[B3] Ellis M, Davis N, Coop A, Liu M, Schumaker L, Lee RY, Srikanchana R, Russell CG, Singh B, Miller WR (2002). Development and validation of a method for using breast core needle biopsies for gene expression microarray analyses. Clin Cancer Res.

[B4] Perou CM, Sorlie T, Eisen MB, van de Rijn M, Jeffrey SS, Rees CA, Pollack JR, Ross DT, Johnsen H, Akslen LA (2000). Molecular portraits of human breast tumours. Nature.

[B5] Sørlie T, Perou CM, Tibshirani R, Aas T, Geisler S, Johnsen H, Hastie T, Eisen MB, van de Rijn M, Jeffrey SS (2001). Gene expression patterns of breast carcinomas distinguish tumor subclasses with clinical implications. Proc Natl Acad Sci USA.

[B6] Sørlie T, Tibshirani R, Parker J, Hastie T, Marron JS, Nobel A, Deng S, Johnsen H, Pesich R, Geisler S (2003). Repeated observation of breast tumor subtypes in independent gene expression data sets. Proc Natl Acad Sci USA.

[B7] van 't Veer LJ, Dai H, van de Vijver MJ, He YD, Hart AA, Mao M, Peterse HL, van der Kooy K, Marton MJ, Witteveen AT (2002). Gene expression profiling predicts clinical outcome of breast cancer. Nature.

[B8] West M, Blanchette C, Dressman H, Huang E, Ishida S, Spang R, Zuzan H, Olson JA, Marks JR, Nevins JR (2001). Predicting the clinical status of human breast cancer by using gene expression profiles. Proc Natl Acad Sci USA.

[B9] Whitney AR, Diehn M, Popper SJ, Alizadeh AA, Boldrick JC, Relman DA, Brown PO (2003). Individuality and variation in gene expression patterns in human blood. Proc Natl Acad Sci USA.

[B10] Tibshirani R, Hastie T, Narasimhan B, Chu G (2002). Diagnosis of multiple cancer types by shrunken centroids of gene expression. Proc Natl Acad Sci USA.

[B11] Hastie T, Tibshirani R, Friedman J (2001). The Elements of Statistical Learning.

[B12] Ambroise C, McLachlan GJ (2002). Selection bias in gene extraction on the basis of microarray gene-expression data. Proc Natl Acad Sci USA.

[B13] Ceci M, Gaviraghi C, Gorrini C, Sala LA, Offenhauser N, Marchisio PC, Biffo S (2003). Release of elF6 (p27-BBP) from the 60S subunit allows 80S ribosome assembly. Nature.

[B14] Ahmad K, Henikoff S (2002). Histone H3 variants specify modes of chromatin assembly. Proc Natl Acad Sci USA.

[B15] Orino K, Lehman L, Tsuji Y, Ayaki H, Torti SV, Torti FM (2001). Ferritin and the response to oxidative stress. Biochem J.

[B16] Nisapakultorn K, Ross KF, Herzberg MC (2001). Calprotectin expression inhibits bacterial binding to mucosal epithelial cells. Infect Immun.

[B17] Nicodemus CF, Avraham S, Austen KF, Purdy S, Jablonski J, Stevens RL (1990). Characterization of the human gene that encodes the peptide core of secretory granule proteoglycans in promyelocytic leukemia HL-60 cells and analysis of translated product. J Biol Chem.

[B18] Zhang X, Kluger Y, Nakayama Y, Poddar R, Whitney C, DeTora A, Weismann SM, Newburger PE (2004). Gene expression in mature neutrophils: early responses to inflammatory stimuli. J Leukoc Biol.

[B19] Subrahmanyam YV, Yamga S, Prashar Y, Lee HH, Hoe NT, Kluger Y, Gerstein M, Goguen JD, Newburger PE, Weismann SM (2001). RNA expression patterns change dramatically in human neutrophils exposed to bacteria. Blood.

