# Supplementary figures and images for: Early detection of breast cancer based on gene-expression patterns in peripheral blood cells
Source: Breast Cancer Res. 2005 Jun 14;7(5):R634–44. doi: 10.1186/bcr1203 (PMC1242124; doi:10.1186/bcr1203)

# Supplemental **Figure 1**

Before batch adjustment

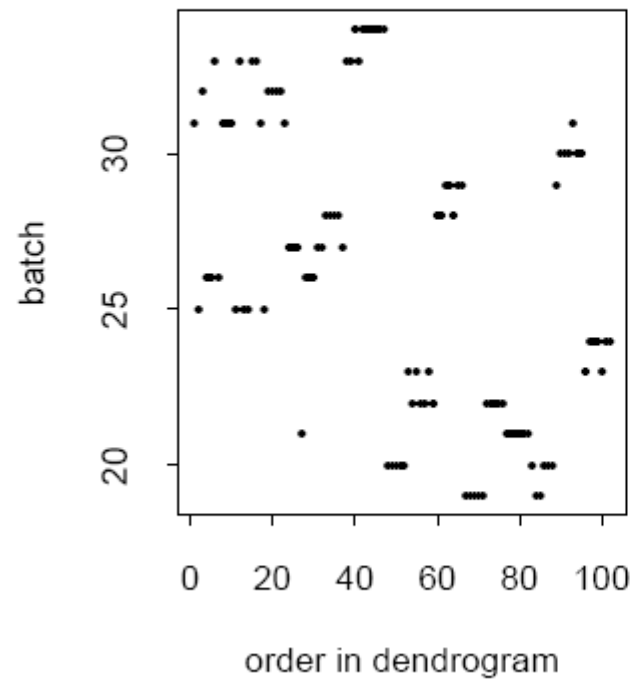

After batch adjustment

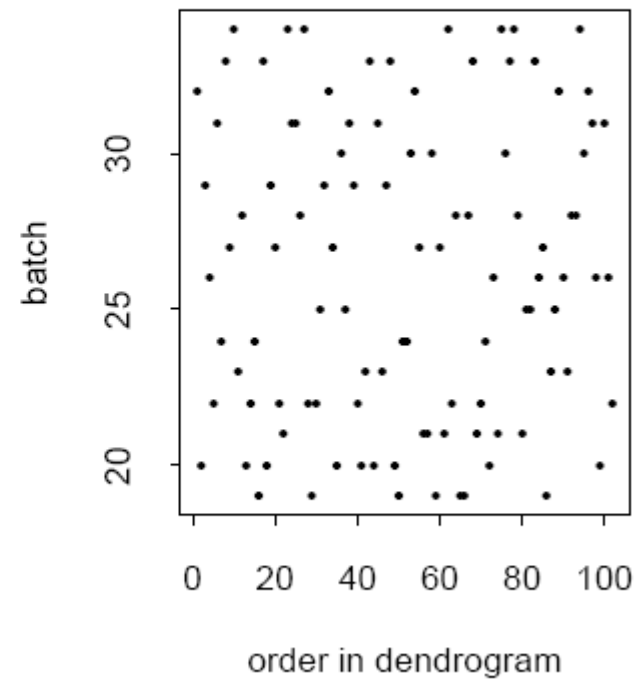

Supplement: Additional File 1 — Supplementary Figure 1, a pdf showing batch adjustment. (Left) Normalized data before batch adjustment; (right) normalized data after batch adjustment by ANOVA. [file bcr1203-S1.pdf]

# Supplemental Figure 2

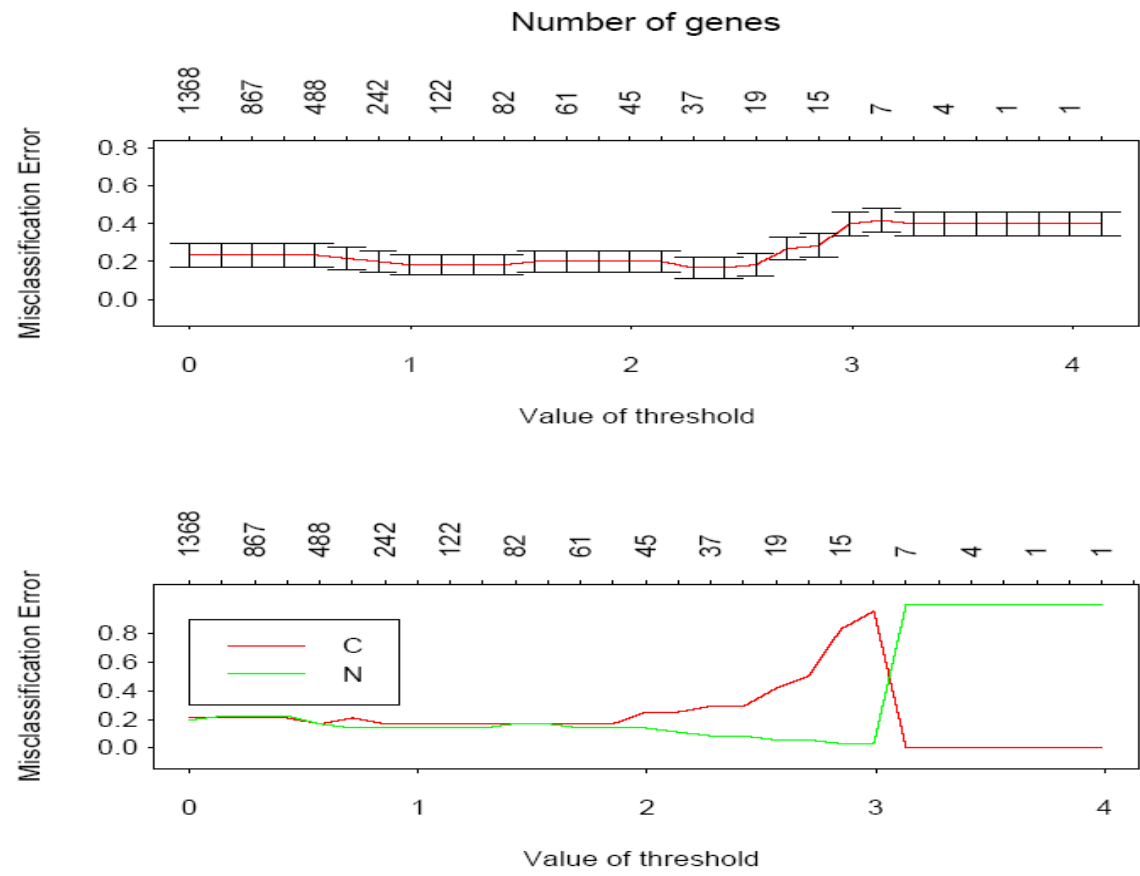

Supplement: Additional File 4 — Supplementary Figure 2, pdf showing misclassification rate as a function of threshold value and the number of genes involved when the error is calculated by taking an average of the class probability for each sample in all 60 cross-validation segments. The upper graph shows that the minimum overall misclassification error is observed at a threshold value of 2.42. The lower graph shows the profile for the misclassification error for breast-cancer (C) and non-breast-cancer (N) samples as a function of threshold value and the number of genes involved. [file bcr1203-S4.pdf]

Supplemental **Figure 3**

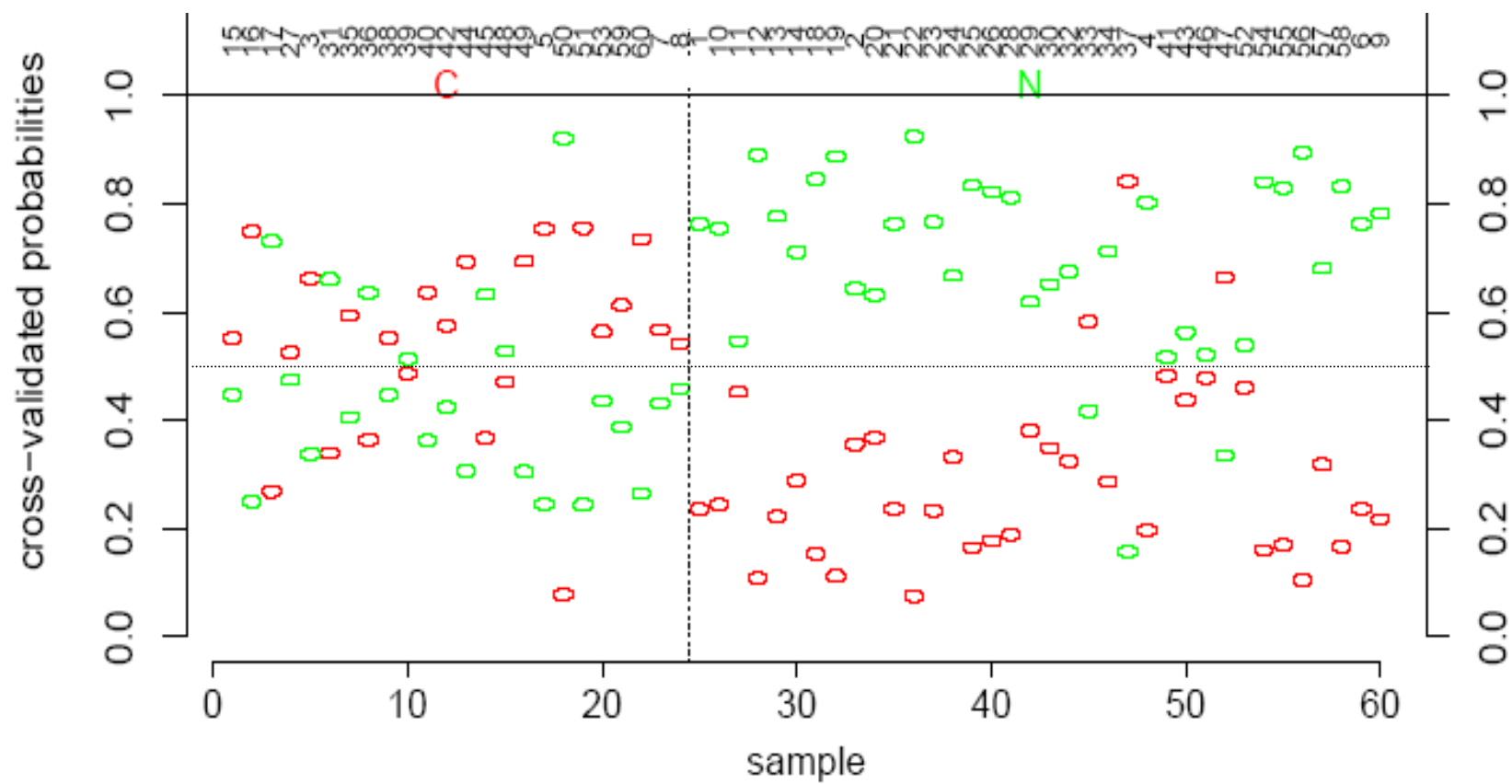

Supplement: Additional File 5 — Supplementary Figure 3, a pdf showing estimated cross-validated probabilities of 60 different blood samples. Red circles represent breast-cancer class (C) and green circles represent non-breast-cancer class (N). Each sample has two probabilities, one for the breast-cancer class and the other for the non-breast-cancer class. The sample is classified in the class whose probability is >0.5. [file bcr1203-S5.pdf]
